# Enhancing Health Service Delivery to Care for Our Aging Population and Their Caregivers: Comment on "Developing a Conceptual Framework for an Age-Friendly Health System: A Scoping Review"

**DOI:** 10.34172/ijhpm.9569

**Published:** 2026-04-15

**Authors:** Jill I. Cameron

**Affiliations:** Department of Occupational Science and Occupational Therapy, Temerty Faculty of Medicine, University of Toronto, Toronto, ON, Canada.

**Keywords:** Aging, Health Services, Integrated Care, Family Centered Care

## Abstract

Karami and colleagues’ scoping review proposes a conceptual framework for age-friendly health systems based on Van Olmen’s 10-element model. The scoping review mapped existing literature on health service delivery for older adults using Arksey and O’Malley’s methodology. They generated a framework that prioritizes person- and family-centered care to reduce harm, improve satisfaction, and enhance value. Key components include strong governance, trained multidisciplinary teams, integrated service delivery across settings, and active involvement of older adults and caregivers in decision-making. The framework aligns with existing age-related models like PRISMA (Program of Research to Integrate the Services for the Maintenance of Autonomy) and the Universal Model of Family-Centered Care. Future research should focus on operationalizing and implementing core components of Karami’s framework. Co-design is an emerging methodological approach used to develop models of care. It can be used to formally engage older adults, families, and professionals to operationalize core components of Karami’s framework with the goal of improving health service delivery for our aging population.

 With global populations aging at an unprecedented rate, health systems must adapt to address the increasingly complex needs of older adults. Delivering care that is not only effective but also person- and family-centered requires innovative frameworks that build on existing models and incorporate the lived experiences of aging individuals and their caregivers. The scoping review by Karami et al^1^ responds to this challenge by proposing a conceptual framework for an age-friendly health system, grounded in Van Olmen’s health system model.^2^ By defining age-friendly health systems as those that deliver optimal care with reduced harm, increased satisfaction, and improved value, the review contributes meaningfully to the ongoing transformation of health systems to better serve aging populations.

 The review used Van Olmen’s health system model,^2^ as the foundational structure for analyzing the literature. This model, applicable at local, regional, and national levels, identifies ten core elements essential to health systems including goals and outcomes, values and principles, service delivery, context, leadership and governance, and the organization of resources (eg, finances, human resources, infrastructure, information).^2^ In the review, data were extracted and summarized based on these dimensions. This structured approach is a strength of the review as it allowed the authors to systematically map the existing literature on core elements present in existing health service delivery models for the elderly.

 The research team employed the Arksey and O’Malley scoping review methodology^3^; however, several elements of the methodological approach require further clarification. In particular, the definitions of population, concept, and context appear to reflect the intended outcomes of the review rather than serving as criteria to guide article selection. For example, the population was defined as “studies assessing each dimension of an age-friendly health system”^1^ (page 2), whereas a more conventional framing would define the population as older adults, the concept as models of health service delivery, and the context as global. The framing used in the review raises the possibility that studies addressing only some dimensions of an age-friendly health system may have been inadvertently excluded. Although the authors describe the overall scoping review process, the types of studies included remain unclear. Specifically, it is uncertain whether the review was limited to articles describing health service delivery models for older adults or whether studies addressing older adults’ preferences and experiences with health services were also included. Greater transparency regarding the inclusion criteria and types of eligible studies would facilitate interpretation of the findings. In addition, the review included a consultation phase in which experts and practitioners in aging-related health services were presented with a conceptual framework; however, the methods used to develop this framework are not described. Explicitly outlining the process by which the conceptual framework was constructed would enhance methodological transparency and strengthen confidence in the review’s conclusions.

 The findings of the review highlight the core components of health service delivery models for older adults that can serve as the foundation for designing comprehensive models in the future. The review underscores the critical importance of establishing governance structures within health services that are specifically designed to prioritize the unique needs of older adults.^1^ These structures should actively promote the inclusion of older individuals, their families and caregivers in treatment decision-making processes, ensuring their voices are heard and respected.^1^ A key focus is on human resources, emphasizing the need for healthcare professionals to be adequately trained to care for an aging population. This includes working collaboratively within multidisciplinary teams, engaging in continuous professional development, and adopting strategies that reduce the financial burden of care on older adults. The review also advocates for the creation of elderly-friendly care environments and the consistent use of evidence-based practices to meet the complex and evolving needs of older patients.^1^

 In terms of service delivery, the review highlights the necessity of providing care across diverse environments, integrating services to ensure continuity and accessibility.^1^ It stresses the importance of involving not only older adults but also their families and caregivers in care planning and decision-making, thereby fostering a more holistic and person-centered approach. Reducing barriers to access and improving the overall quality of services are identified as essential goals.^1^ The outcomes of implementing an age-friendly health system include improved health results for older individuals, the prevention of avoidable healthcare-related harms, and the delivery of high-quality, satisfying care that supports autonomy and self-care.^1^ Furthermore, the review points to the need for robust communication and information-sharing mechanisms between institutions to facilitate effective service planning and coordination.^1^ It also calls for the active involvement of multiple knowledge users, ranging from policy-makers to community organizations, in shaping a health system that is responsive to the needs of older adults. Finally, the physical and social environments within healthcare settings must be adapted to be age-friendly, ensuring that they are safe, accessible, and conducive to the well-being of elderly patients.^1^

 The conceptual framework proposed by Karami et al^1^ offers a valuable foundation for enhancing health service delivery for the aging population. Future research should focus on operationalizing the framework’s components and can build upon existing research not included in the review. Karami’s framework recommends identifying concrete strategies for integrating services across various providers and care environments. The PRISMA (Program of Research to Integrate the Services for the Maintenance of Autonomy) model for the elderly originating in Québec, Canada, is an integrated, coordination-focused service delivery model tailored for frail older adults that has developed strategies to achieve this goal.^4^ Its core components include coordination mechanisms between decision-makers, managers, and providers; a single point of entry to simplify access; and dedicated case management where a case manager oversees the aging individual’s care journey. The model features comprehensive assessment, individualized care plans, and enhanced communication across institutions and providers using a computerized clinical chart. Governance is organized across strategic, tactical, and operational levels, with local governance tables, steering committees, and multidisciplinary teams supporting implementation. Preliminary outcomes show improved continuity, reduced functional decline, heightened patient and caregiver satisfaction and empowerment, and reduced emergency visits.^4^

 Karami’s framework recommends involving families and caregivers in care planning and decision-making.^1^ Although the framework does not emphasize the importance of family members in the care of the elderly, family caregivers play an important role contributing to the sustainability of healthcare systems. For example, the unpaid labour provided by caregivers in Canada annually saves the healthcare system over $90 billion dollars.^5^ To enhance the inclusion of family caregivers in health service delivery models for the elderly, future research can build upon a previous review that defined a Universal Model of Family-Centered care.^6^ The review synthesized models from various age groups and clinical contexts.^6^ The central goal of these models is to develop and implement patient care plans that are explicitly created with and within the family context, fostering partnerships and co-ownership of health outcomes. Their analysis of 55 diverse models revealed four core, universally applicable components: (1) collaboration between patients, family members, and healthcare providers; (2) consideration of family context, including the needs, roles, and circumstances of caregivers; (3) policies and procedures formalizing family involvement in care delivery; and (4) education targeting patients, families, and healthcare professionals to support shared decision-making.^6^ While some elements remain illness-specific (eg, condition-tailored education), these four elements form the foundation of family-centered care across diverse populations and care settings. This model can be used to operationalize the person and family engagement in care planning and decision-making for the care of our aging population.

 As research progresses to operationalize Kamari’s conceptual framework, the emerging practice of co-design presents a promising avenue for involving end users, including older adults, their families, caregivers, and healthcare professionals, in the design of both models and services, to make systems more responsive to their lived experiences and needs. Co-design is a collaborative approach to designing services, systems, or models that actively involves end users throughout the development process.^7,8^ In practice, co-design typically begins with identifying the needs and experiences of users through interviews, focus groups, or workshops. For example, the concepts included in Karami’s framework, could be explored with older adults, caregivers, and healthcare professionals to begin to capture their preferences related to age-friendly service deliver models. Knowledge users can use the framework and the information gathered to work together to brainstorm solutions, test prototypes, and evaluate outcomes, to make a final product that reflects real-world needs and preferences. In healthcare, co-design helps create more person- and family-centered and responsive services by embedding the lived experiences of older adults and their support networks into the design of care models, environments, and delivery strategies. This method fosters ownership, relevance, and sustainability of the solutions developed.

 A key strength of the conceptual framework proposed by Karami et al is its foundation in Van Olmen’s health system model, which enabled the review to build upon established knowledge while expanding it through specific examples for each domain drawn from the literature.^1,2^ The inclusion of a consultation phase added further value by formally integrating the perspectives of researchers and knowledge users, enriching the framework’s relevance and applicability. It emphasizes the importance of interdisciplinary care involving professionals with expertise in geriatric health and highlights the necessity of creating age-friendly care environments that support the dignity, safety, and comfort of older adults. Notably, the model is designed to address the needs of not only elderly patients but also their families and caregivers, aligning with family-centered care approaches such as those described by Kokorelias et al.^6^

 In summary, the review by Karami et al contributes to advancing health service delivery for the aging population by proposing a conceptual framework grounded in Van Olmen’s health system model.^1,2^ This foundation enabled the review to build upon and expand existing concepts, offering a structured approach to understanding and improving care for older adults. The proposed framework aligns with established models such as Hébert’s PRISMA model,^4^ which emphasizes integrated service delivery through case management, and the Universal Model of Family-Centered care,^6^ which highlights collaboration and the inclusion of family and caregivers in care planning and decision-making. Together, these alignments reinforce the relevance and applicability of Karami and colleagues’ framework. However, future research incorporating co-design methods that engage end users in shaping care systems is needed to operationalize the framework’s components.

## Disclosure of artificial intelligence (AI) use

 The author discloses that AI was used in the editing process to improve awkwardly written sentences.

## Ethical issues

 Not applicable.

## Conflicts of interest

 Author declares that she has no conflicts of interest.
